# Portal hypertension mortality in the United States (1999–2023): Insights from the CDC WONDER database

**DOI:** 10.1097/MD.0000000000050127

**Published:** 2026-08-07

**Authors:** Raymond Haward, Nikhil Duseja, Het Hirpara, Harkanwar Sandhu, Swathi Deepak Kulkarni, Rachel Haward, Mobeen Abid, Mir Mehdi Hussain, Newton Ashish Shah, Iswariya Mani, Pooja Prasad, Jai Kumar Rajavoor Muniswamy

**Affiliations:** a Vydehi Institute of Medical Sciences & Research Centre, Bengaluru, India; b Karachi Medical and Dental College, Karachi, Pakistan; c C.U.Shah Medical College and Hospital, Surendranagar, India; d Christian Medical College and Hospital, Vellore, India; e HCA Healthcare/Tuft School of Medicine: Portsmouth Regional Hospital, Portsmouth, NH; f K.V.G. Medical College & Hospital, Sullia, India; g Tribhuvan University Teaching Hospital, Kathmandu, Nepal; h UPMC Medical Educational Program, Pittsburgh, PA; i Johns Hopkins Bloomberg School of Public Health, Baltimore, MD; j Mayo clinic, Jacksonville, FL.

**Keywords:** gastroenterology, mortality, portal hypertension

## Abstract

Portal hypertension is a central driver of morbidity and mortality in advanced chronic liver disease, yet its contribution to death is frequently underestimated owing to underreporting on death certificates. Population-level data on portal hypertension-related mortality trends and disparities in the United States remain limited. We conducted a retrospective, population-based mortality study using the Centers for Disease Control and Prevention Wide-ranging Online Data for Epidemiologic Research Multiple Cause of Death database, identifying deaths with International Statistical Classification of Diseases and Related Health Problems, 10th Revision code K76.6 listed in any cause-of-death field from 1999 to 2023. Crude and age-adjusted mortality rates per 100,000 population were calculated and stratified by sex, U.S. Census region, race/ethnicity, and urban–rural status (the latter restricted to 1999–2020 due to data availability). Temporal trends were assessed using Joinpoint regression, with annual percent change (APC) reported as the primary trend metric and 95% confidence intervals presented for all estimates. A total of 34,551 portal hypertension-related deaths were identified. Overall age-adjusted mortality rates declined sharply from 1999 to 2002 (APC − 12.85%), plateaued for more than a decade, and rose thereafter. Mortality increased significantly in women after 2009 (APC + 6.69%) and in men after 2013 (APC + 6.18%). Among racial and ethnic groups, Hispanic or Latino individuals consistently demonstrated the highest mortality burden throughout the study period, followed by White individuals, whereas Black or African American individuals exhibited comparatively lower but significantly rising rates from 2006 onward. White individuals demonstrated the steepest recent acceleration, with a significant increase after 2014 (APC + 8.51%). Regionally, the South experienced the steepest post-2013 increase, while the Northeast retained the lowest absolute rates despite a significant rise after 2014. Urban populations showed early improvement through 2014 followed by rising mortality, whereas rural populations experienced a later but steeper rise beginning around 2013, with widening urban–rural disparities over time. Portal hypertension-related mortality has risen significantly since the mid-2010s across all demographic and geographic subgroups, with disproportionate burdens among Hispanic individuals, men, rural populations, and residents of the Southern United States. These findings highlight the urgent need for targeted prevention strategies, equitable access to hepatology care, and aggressive management of metabolic and alcohol-related risk factors to reverse this trajectory.

## 1. Introduction

Portal hypertension is a hallmark of advanced chronic liver disease and the central pathophysiologic driver of its most serious complications, including esophagogastric variceal bleeding, ascites, and hepatic encephalopathy.^[1,2]^ Once portal hypertension develops, it initiates a cascade of hemodynamic and systemic disturbances that accelerates the transition from compensated to decompensated cirrhosis and substantially worsens prognosis.^[1,2]^ Together, these complications account for a large share of morbidity and mortality in cirrhosis, with variceal hemorrhage among the leading causes of death in this population.^[3]^ Clinically significant portal hypertension affects approximately 0.98% of U.S. adults, and 30 to 50% of individuals with compensated cirrhosis develop esophagogastric varices, underscoring its early and widespread clinical impact.^[4]^ Despite this burden, portal hypertension is consistently underrepresented in mortality statistics that rely solely on the underlying cause of death, because deaths are typically attributed to its downstream complications rather than to portal hypertension itself.^[5,6]^

Liver-related mortality in the United States has risen substantially over the past 2 decades, reflecting a growing and inadequately addressed public health concern. National analyses of Centers for Disease Control and Prevention mortality data from 1999 to 2023 demonstrate a nearly doubling of annual liver disease deaths, from approximately 50,524 to 99,217, with age-adjusted mortality increasing from 28.49 to 36.71 per 100,000 population.^[7]^ Alcohol-associated liver disease and primary liver cancer now account for more than half of liver-related deaths, mortality from viral hepatitis has declined following the introduction of curative therapy for hepatitis C, and deaths attributable to metabolic dysfunction-associated steatotic liver disease continue to rise.^[7,8]^ Marked demographic and geographic disparities persist, with higher and more rapidly increasing mortality among Hispanic adults, adults aged 25 to 34 years, women, and residents of nonmetropolitan areas in the South and West.^[7,9]^ As the central hemodynamic driver of decompensation across these etiologies, portal hypertension contributes to the majority of liver-related deaths, yet its specific contribution to national mortality trends remains inadequately characterized.^[4]^

Despite its clinical importance, nationally representative, long-horizon data describing the mortality burden of portal hypertension in the United States remain limited and methodologically challenging to obtain, in large part because most prior analyses have relied on underlying-cause-of-death coding or single-center cohorts.^[5,10]^ To address this gap, we used the Centers for Disease Control and Prevention Wide-ranging Online Data for Epidemiologic Research (CDC WONDER) Multiple Cause of Death (MCD) database to perform a comprehensive, nationally representative analysis of portal hypertension-associated mortality from 1999 to 2023. We hypothesized that portal hypertension-associated mortality has increased over time in parallel with the rising burden of chronic liver disease, that meaningful demographic and geographic disparities exist by sex, U.S. Census region, Race/ethnicity and urban–rural status, and that mortality patterns have evolved in step with shifting etiologies of advanced liver disease.^[11,12]^

## 2. Methodology

### 2.1. Study setting and population

This study is a retrospective mortality review of deaths associated with portal hypertension across the United States from 1999 to 2023. Data were obtained from the CDC WONDER MCD database, a publicly available and authoritative source that includes death certificate records from all 50 states and the District of Columbia. Unlike analyses restricted to the underlying cause of death, the MCD database captures portal hypertension whenever it is recorded anywhere on the death certificate, whether as the primary cause or as a contributing condition, thereby providing a more comprehensive ascertainment of portal hypertension associated mortality. Urbanization data were available from 1999 to 2020 only from the database. 2021 to 2023 data were included in the overall trend analysis but excluded from urban–rural stratified analysis due to the unavailability of classification from 2021 to 2023. Race/ethnicity data were available across the full study period (1999–2023) and were included in all stratified analyses. Causes of death were identified using the International Statistical Classification of Diseases and Related Health Problems, 10th Revision (ICD-10), with ICD-10 code K76.6 for portal hypertension. This MCD approach was chosen given that portal hypertension is frequently under-documented as the underlying cause of death, with mortality more commonly attributed to its downstream complications such as variceal hemorrhage, hepatorenal syndrome, and hepatic encephalopathy. Since the dataset is publicly available and de-identified, it did not require Institutional Review Board review.

### 2.2. Data extraction

We extracted data on annual mortality, sex-stratified mortality, race/ethnicity-stratified mortality, and geographic variation (by U.S. Census region and urban–rural classification) in portal hypertension-related deaths across all age groups from 1999 to 2023; urban–rural analyses were restricted to 1999 to 2020 due to data availability. Decedents were included if ICD-10 code K76.6 was listed in any cause-of-death field, capturing both underlying and contributing causes. All data were queried and extracted from the CDC WONDER MCD online interface in June 2025. Queries were performed using ICD-10 code K76.6 applied across all cause-of-death fields, with grouping by year, sex, race/ethnicity, U.S. Census region, and urban–rural classification. In accordance with CDC WONDER data use guidelines, death counts based on fewer than 10 events are suppressed to protect privacy; any stratum yielding a suppressed count was excluded from rate calculations and clearly noted. No imputation was performed for suppressed cells. Demographic and geographic classifications were derived from death-certificate data, consistent with prior analyses using the CDC WONDER database. Sex was recorded as male or female, and race/ethnicity was categorized into 3 groups as recorded in the CDC WONDER database: White, Black or African American, and Hispanic or Latino. Race/ethnicity classifications were derived from death-certificate data and are subject to known limitations regarding misclassification. Decedents not classifiable into 1 of these 3 groups were excluded from race/ethnicity-stratified analyses. Decedents were assigned to 1 of 4 U.S. Census regions: Midwest, Northeast, South, and West. Urbanization was characterized using the 2013 National Center for Health Statistics Urban–Rural Classification Scheme, which assigns counties to 6 categories (Large Central Metro, Large Fringe Metro, Medium Metro, Small Metro, Micropolitan, and NonCore). For analysis, these were collapsed into urban (Large Central, Large Fringe, Medium, and Small Metro) and rural (Micropolitan and NonCore) strata. Urbanization analyses were restricted to 1999 to 2020, as county-level urban–rural classifications are not available in CDC WONDER beyond 2020.

### 2.3. Statistical analysis

Crude and age-adjusted mortality rates (AAMRs) per 100,000 population for portal hypertension-related deaths were calculated for 1999 to 2023 and stratified by year, sex, race/ethnicity, U.S. Census region, and urban–rural status. All estimates are presented with 95% confidence intervals (CIs). Crude rates were calculated as the annual number of qualifying deaths divided by the corresponding U.S. population, and AAMRs were standardized to the year 2000 U.S. standard population using the direct method to permit comparison over time.

Temporal trends in AAMRs were evaluated using Joinpoint regression (Joinpoint Regression Program, version 5.2.0.0; National Cancer Institute). Joinpoint analyses were performed for the overall cohort and separately for each race/ethnicity group to characterize group-specific trend inflection points. Log-linear models were fit with a minimum of 0 and a maximum of 4 joinpoints, consistent with the program’s recommendation of approximately 1 joinpoint per 5 to 7 annual data points for a 25-year series. The optimal model was selected by the permutation test method with an overall significance level of α = 0.05 (Bonferroni-adjusted across joinpoint comparisons) and a grid search across all candidate joinpoint locations. To account for heteroscedasticity, standard errors of the AAMRs were computed assuming Poisson variation in the death counts, and models were fit by weighted least squares with weights equal to the inverse of the squared standard errors. Models were estimated assuming uncorrelated errors; a sensitivity analysis using the data-dependent autocorrelated-errors option produced essentially identical results, indicating no material residual autocorrelation. The annual percent change (APC) with 95% CI was estimated for each identified trend segment and served as the primary trend metric, and the average APC was reported as a secondary summary measure for the overall 1999 to 2023 period. Two-sided *P* < .05 was considered statistically significant.

Sensitivity analyses were performed to assess the robustness of our findings. First, mortality estimates based on multiple-cause-of-death data were compared with those restricted to portal hypertension as the underlying cause; the latter yielded lower absolute rates but comparable temporal trends. Second, crude and age-adjusted rates were compared and showed consistent trajectories, indicating that observed trends were not driven by population aging or case definition alone. Third, race/ethnicity-stratified analyses were examined for strata with small death counts, and any group-year combinations yielding suppressed counts were excluded from trend modeling to avoid unstable estimates. Fourth, Joinpoint models were reestimated across varying numbers of permitted joinpoints and under both uncorrelated- and autocorrelated-error specifications to confirm that identified inflection points were stable and not artifacts of overfitting or error-structure assumptions. All analyses followed standard practices for population-level mortality trend studies.

### 2.4. Protocol approval and patient consent

This analysis used publicly available, de-identified mortality data obtained from the CDC WONDER database. As no identifiable patient-level information was accessed, the study was exempt from Institutional Review Board review and informed consent requirements in accordance with federal human subjects research guidelines.

## 3. Results

**Figure 1.**
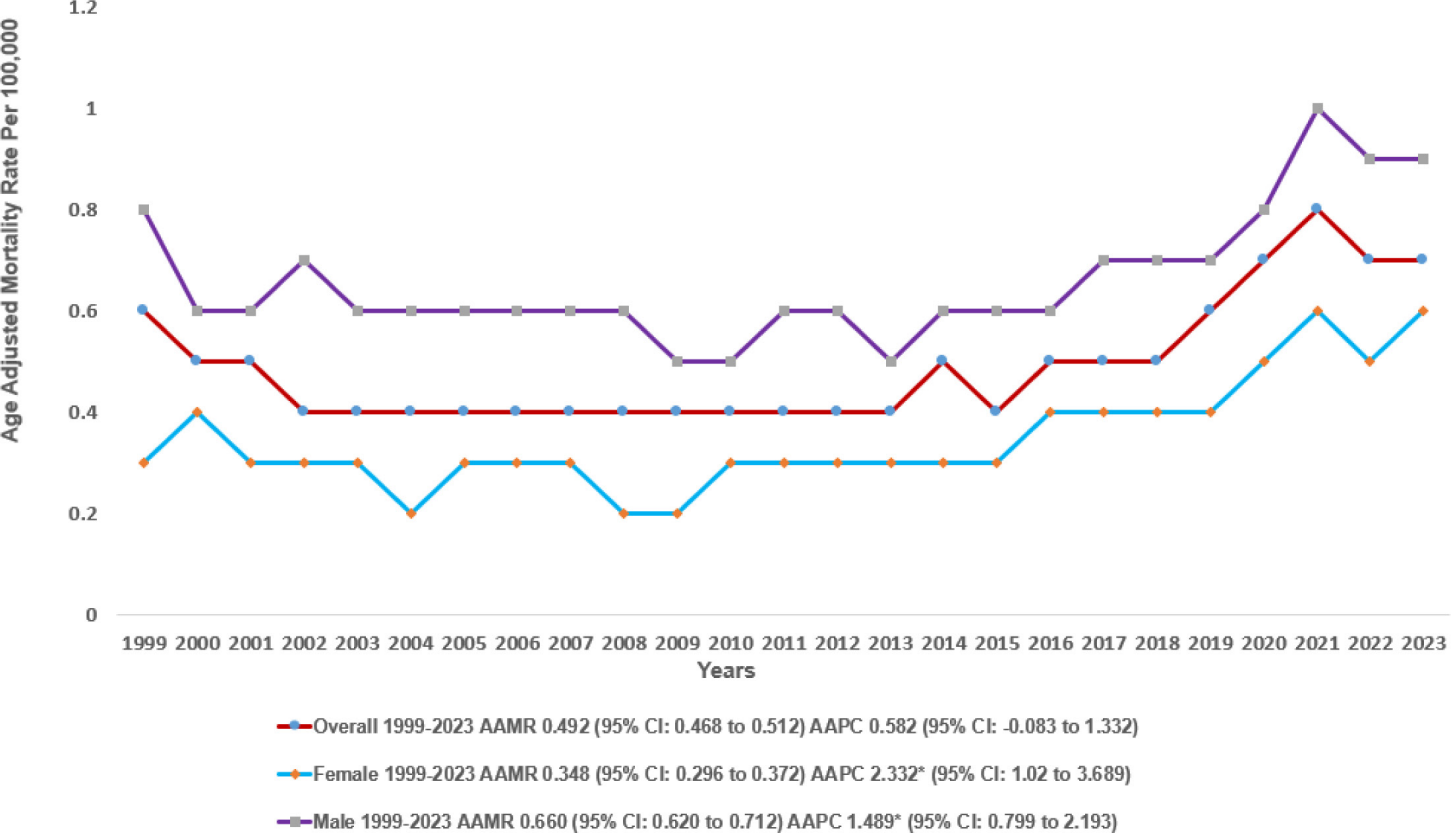
Overall and gender-stratified portal hypertension age-adjusted mortality rates per 100,000 adults in the United States, 1999–2023. AAMR = age-adjusted mortality rate, AAPC = average annual percent change, CI = confidence interval.

From 1999 to 2023, a total of 34,551 deaths were attributed to portal hypertension in the United States. The AAMR declined significantly from 1999 to 2002 (0.6 to 0.4; APC = −12.85; 95% CI: −20.89 to −7.50), followed by a period of relative stability from 2002 to 2015 (APC = 0.39; 95% CI: −0.53 to 2.09; Tables 1 and 2; Fig. 1). Mortality subsequently increased from 2015 to 2021 (0.4 to 0.8; APC = 10.28; 95% CI: −0.97 to 18.08), followed by a nonsignificant decline from 2021 to 2023 (0.8 to 0.7; APC = −4.21; 95% CI: −10.74 to 7.29).

**Table 1:** Total deaths and sex-stratified mortality associated with portal hypertension in the United States, 1999–2023.

Year	Population	Deaths (Overall)	Deaths (Male)	Deaths (Female)
1999	219,084,800	1134	739	395
2000	221,168,531	1086	665	421
2001	224,518,698	1031	658	373
2002	227,062,163	1033	698	335
2003	229,479,283	1015	650	365
2004	232,153,496	956	628	328
2005	234,997,553	1000	661	339
2006	237,863,203	1033	672	361
2007	240,549,592	1076	697	379
2008	243,186,582	1028	687	341
2009	245,683,948	1034	676	358
2010	247,518,325	1068	676	392
2011	250,390,811	1152	776	376
2012	252,769,942	1178	771	407
2013	255,039,716	1174	722	452
2014	257,789,101	1306	832	474
2015	260,402,033	1338	847	491
2016	262,152,444	1514	906	608
2017	264,697,626	1585	1003	582
2018	266,281,990	1668	1062	606
2019	267,668,677	1766	1094	672
2020	269,190,697	2133	1272	861
2021	271,327,075	2461	1453	1008
2022	273,850,170	2357	1443	914
2023	275,416,414	2425	1467	958

**Table 2:** Age-adjusted mortality rates (AAMRs) with 95% confidence intervals for portal hypertension in the United States, stratified by sex (male and female), 1999–2023.

Year	Overall AAMR (95% CI)	Male AAMR (95% CI)	Female AAMR (95% CI)
1999	0.6 (0.5–0.6)	0.8 (0.7–0.8)	0.3 (0.3–0.4)
2000	0.5 (0.5–0.5)	0.6 (0.6–0.7)	0.4 (0.3–0.4)
2001	0.5 (0.4–0.5)	0.6 (0.6–0.7)	0.3 (0.3–0.3)
2002	0.4 (0.4–0.5)	0.7 (0.6–0.7)	0.3 (0.2–0.3)
2003	0.4 (0.4–0.4)	0.6 (0.6–0.7)	0.3 (0.2–0.3)
2004	0.4 (0.4–0.4)	0.6 (0.5–0.6)	0.2 (0.2–0.3)
2005	0.4 (0.4–0.4)	0.6 (0.5–0.6)	0.3 (0.2–0.3)
2006	0.4 (0.4–0.5)	0.6 (0.5–0.6)	0.3 (0.2–0.3)
2007	0.4 (0.4–0.4)	0.6 (0.6–0.7)	0.3 (0.3–0.3)
2008	0.4 (0.4–0.4)	0.6 (0.5–0.6)	0.2 (0.2–0.3)
2009	0.4 (0.4–0.4)	0.5 (0.5–0.6)	0.2 (0.2–0.3)
2010	0.4 (0.4–0.4)	0.5 (0.5–0.6)	0.3 (0.2–0.3)
2011	0.4 (0.4–0.4)	0.6 (0.5–0.6)	0.3 (0.2–0.3)
2012	0.4 (0.4–0.5)	0.6 (0.5–0.6)	0.3 (0.2–0.3)
2013	0.4 (0.4–0.4)	0.5 (0.5–0.6)	0.3 (0.3–0.3)
2014	0.5 (0.4–0.5)	0.6 (0.6–0.6)	0.3 (0.3–0.3)
2015	0.4 (0.4–0.5)	0.6 (0.6–0.6)	0.3 (0.3–0.3)
2016	0.5 (0.5–0.5)	0.6 (0.6–0.7)	0.4 (0.3–0.4)
2017	0.5 (0.5–0.5)	0.7 (0.6–0.7)	0.4 (0.3–0.4)
2018	0.5 (0.5–0.5)	0.7 (0.7–0.8)	0.4 (0.3–0.4)
2019	0.6 (0.5–0.6)	0.7 (0.7–0.8)	0.4 (0.4–0.4)
2020	0.7 (0.6–0.7)	0.8 (0.8–0.9)	0.5 (0.5–0.6)
2021	0.8 (0.7–0.8)	1.0 (0.9–1.0)	0.6 (0.5–0.6)
2022	0.7 (0.7–0.7)	0.9 (0.9–1.0)	0.5 (0.5–0.6)
2023	0.7 (0.7–0.8)	0.9 (0.9–1.0)	0.6 (0.5–0.6)

AAMRs = age-adjusted mortality rates, CI = confidence interval.

Overall, mortality increased in both sexes, with a greater relative rise among females (0.3 to 0.6; APC = 2.33; 95% CI: 1.02 to 3.69) compared to males (0.8 to 0.9; APC = 1.49; 95% CI: 0.80 to 2.19). Among females, mortality declined from 1999 to 2009 (APC = −3.47; 95% CI: −12.57 to 0.05), followed by a significant increase from 2009 to 2023 (0.2 to 0.6; APC = 6.69; 95% CI: 4.33 to 11.69; Tables 1 and 2; Fig. 1). In males, mortality declined significantly from 1999 to 2013 (0.8 to 0.5; APC = −1.73; 95% CI: −3.56 to −0.51), followed by a significant increase from 2013 to 2023 (0.5 to 0.9; APC = 6.18; 95% CI: 4.03 to 9.62).

**Figure 2.**
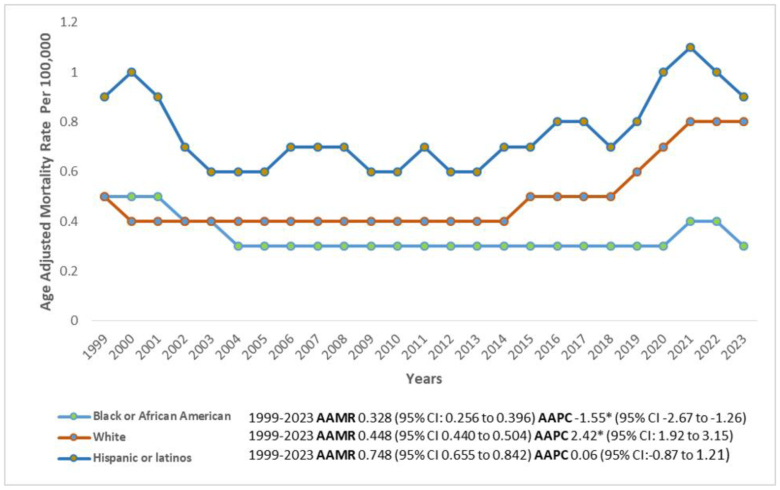
Race/ethnicity-stratified portal hypertension age-adjusted mortality rates per 100,000 adults in the United States, 1999–2023. AAMR = age-adjusted mortality rate, AAPC = average annual percent change, CI = confidence interval.

Ethnicity-specific trends showed that Black or African Americans experienced a significant decline from 1999 to 2006 (0.5 to 0.3; APC = −8.64; 95% CI: −14.46 to −5.67), followed by a significant but modest increase from 2006 to 2023 (0.3 to 0.3; APC = 0.94; 95% CI: 0.03 to 2.13). White individuals experienced a significant decline from 1999 to 2001 (0.5 to 0.4; APC = −9.53; 95% CI: −14.63 to −0.42), followed by a nonsignificant increase from 2001 to 2014 (0.4 to 0.4; APC = 0.31; 95% CI: −0.51 to 6.49), and then a significant increase from 2014 to 2023 (0.4 to 0.8; APC = 8.51; 95% CI: 6.86 to 10.95). Hispanic or Latino individuals experienced a significant decline from 1999 to 2003 (0.9 to 0.6; APC = −10.89; 95% CI: −22.40 to −2.57), followed by a nonsignificant increase from 2003 to 2013 (0.6 to 0.6; APC = 0.02; 95% CI: −5.51 to 8.08), and a further nonsignificant increase from 2013 to 2023 (0.6 to 0.9; APC = 4.87; 95% CI: −2.97 to 15.45; Tables 3 and 4; Fig. 2). Racial and ethnic disparities were evident throughout the study period. Hispanic individuals consistently demonstrated the highest burden of portal hypertension-related mortality, followed by White individuals, whereas Black individuals exhibited comparatively lower rates.

**Table 3:** Overall deaths associated with portal hypertension in the United States, stratified by race/ethnicity (White, Black or African American, and Hispanic or Latino), 1999–2023.

Year	Black or African American		White		Hispanic or latinos	
	Death	Population	Death	Population	Death	Population
1999	110	25,368,995	861	1.6E + 08	128	23,820,423
2000	120	25,680,610	773	1.6E + 08	152	24,800,752
2001	109	26,200,056	739	1.61E + 08	139	26,145,031
2002	99	26,608,232	771	1.62E + 08	122	27,221,488
2003	104	27,004,431	759	1.62E + 08	111	28,267,428
2004	75	27,476,670	722	1.63E + 08	122	29,331,523
2005	90	27,977,184	749	1.64E + 08	121	30,480,453
2006	81	28,479,671	779	1.64E + 08	135	31,660,841
2007	92	28,957,129	794	1.65E + 08	152	32,826,230
2008	88	29,427,226	752	1.66E + 08	155	34,006,088
2009	84	29,870,553	776	1.66E + 08	136	35,164,546
2010	80	30,193,543	789	1.66E + 08	159	36,047,093
2011	98	30,696,102	818	1.67E + 08	177	37,314,879
2012	92	31,144,346	858	1.68E + 08	169	38,153,973
2013	103	31,566,335	838	1.68E + 08	168	39,059,105
2014	90	32,071,041	949	1.68E + 08	208	40,228,555
2015	100	32,540,021	969	1.69E + 08	202	41,290,791
2016	94	32,910,127	1086	1.69E + 08	268	42,032,178
2017	95	33,372,768	1160	1.69E + 08	254	43,329,229
2018	96	33,678,703	1234	1.69E + 08	261	44,164,618
2019	115	34,008,649	1271	1.69E + 08	302	44,910,440
2020	107	34,340,095	1533	1.69E + 08	385	45,771,906
2021	132	33,485,203	1748	1.67E + 08	425	46,990,111
2022	133	33,776,725	1701	1.67E + 08	400	48,188,540
2023	125	34,038,785	1761	1.67E + 08	393	49,528,881

**Table 4:** Age-adjusted mortality rates (AAMRs) with 95% confidence intervals associateed with portal hypertension in the United States, stratified by race/ethnicity, 1999–2023.

Year	Black AAMR (95% CI)	White AAMR (95% CI)	Hispanic or Latino AAMR (95% CI)
1999	0.5 (0.4–0.6)	0.5 (0.5–0.5)	0.9 (0.7–1.1)
2000	0.5 (0.4–0.6)	0.4 (0.4–0.5)	1.0 (0.9–1.2)
2001	0.4 (0.3–0.5)	0.4 (0.4–0.4)	0.9 (0.7–1.0)
2002	0.4 (0.4–0.5)	0.4 (0.4–0.5)	0.7 (0.6–0.9)
2003	0.3 (0.2–0.4)	0.4 (0.4–0.4)	0.6 (0.5–0.8)
2004	0.3 (0.3–0.4)	0.4 (0.4–0.4)	0.6 (0.5–0.8)
2005	0.3 (0.2–0.4)	0.4 (0.4–0.4)	0.6 (0.5–0.7)
2006	0.3 (0.2–0.4)	0.4 (0.4–0.4)	0.7 (0.6–0.8)
2007	0.3 (0.3–0.4)	0.4 (0.4–0.4)	0.7 (0.6–0.8)
2008	0.3 (0.2–0.4)	0.4 (0.3–0.4)	0.7 (0.6–0.8)
2009	0.3 (0.2–0.4)	0.4 (0.4–0.4)	0.6 (0.5–0.7)
2010	0.3 (0.2–0.3)	0.4 (0.4–0.4)	0.6 (0.5–0.7)
2011	0.3 (0.2–0.4)	0.4 (0.4–0.4)	0.7 (0.6–0.8)
2012	0.3 (0.2–0.4)	0.4 (0.4–0.4)	0.6 (0.5–0.7)
2013	0.3 (0.3–0.4)	0.4 (0.4–0.4)	0.6 (0.5–0.7)
2014	0.3 (0.2–0.3)	0.4 (0.4–0.5)	0.7 (0.6–0.8)
2015	0.3 (0.2–0.4)	0.4 (0.4–0.5)	0.7 (0.6–0.8)
2016	0.3 (0.2–0.4)	0.5 (0.5–0.5)	0.8 (0.7–1.0)
2017	0.3 (0.2–0.3)	0.5 (0.5–0.6)	0.8 (0.7–0.9)
2018	0.3 (0.2–0.4)	0.5 (0.5–0.6)	0.7 (0.6–0.8)
2019	0.3 (0.3–0.4)	0.6 (0.5–0.6)	0.8 (0.7–0.9)
2020	0.3 (0.2–0.3)	0.7 (0.6–0.7)	1.0 (0.9–1.1)
2021	0.4 (0.3–0.4)	0.8 (0.8–0.8)	1.1 (1.0–1.2)
2022	0.4 (0.3–0.4)	0.8 (0.7–0.8)	1.0 (0.9–1.1)
2023	0.3 (0.3–0.4)	0.8 (0.8–0.8)	0.9 (0.8–1.0)

AAMRs = age-adjusted mortality rates, CI = confidence interval.

**Figure 3.**
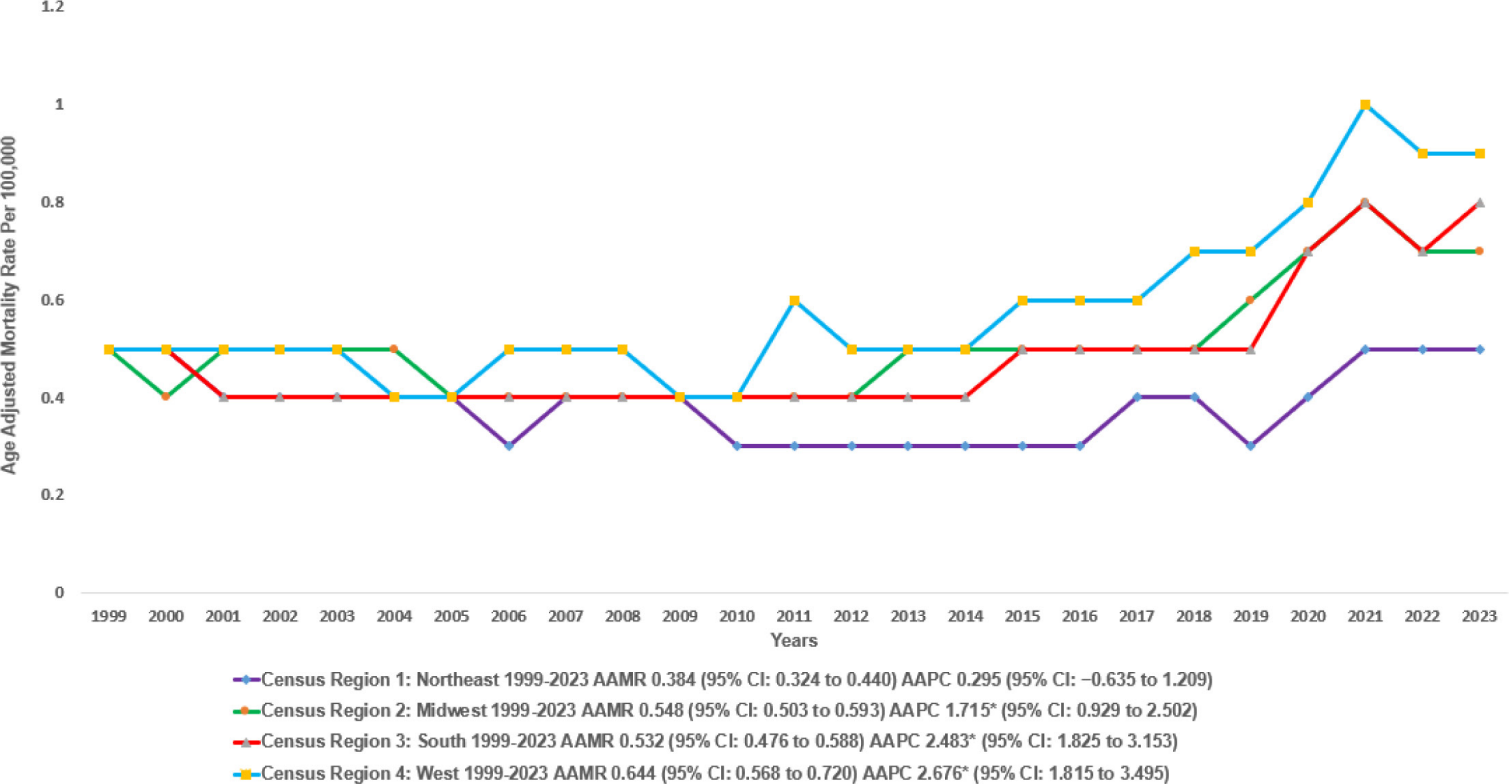
Region-stratified portal hypertension age-adjusted mortality rates per 100,000 adults in the United States, 1999–2023. AAMR = age-adjusted mortality rate, AAPC = average annual percent change, CI = confidence interval.

Regionally, the Northeast demonstrated a significant decline from 1999 to 2014 (0.5 to 0.3; APC = −3.26; 95% CI: −5.43 to −1.82), followed by a significant increase from 2014 to 2023 (0.3 to 0.5; APC = 6.50; 95% CI: 3.24 to 12.57; Tables 5 and 6; Fig. 3). The Midwest showed a similar pattern, with a decline from 1999 to 2010 (0.5 to 0.4; APC = −2.22; 95% CI: −5.89 to −0.31) followed by an increase from 2010 to 2023 (0.4 to 0.7; APC = 5.16; 95% CI: 3.53 to 7.81). In the South, mortality remained relatively stable from 1999 to 2013 (APC = −1.01; 95% CI: −2.64 to 0.19), followed by a significant increase from 2013 to 2023 (0.4 to 0.8; APC = 7.58; 95% CI: 5.49 to 10.74). The West demonstrated a modest decline from 1999 to 2010 (0.5 to 0.4; APC = −1.18; 95% CI: −5.19 to −0.77), followed by a significant increase from 2010 to 2023 (0.4 to 0.9; APC = 6.06; 95% CI: 4.38 to 8.95).

**Table 5:** Overall deaths associated with portal hypertension in the United States, stratified by U.S. Census geographic regions (Northeast, Midwest, South, and West), 1999–2023.

Year	Northeast mortality	Northeast population	Midwest mortality	Midwest population	South mortality	South population	West mortality	West population
1999	233	42,468,049	266	50,314,944	401	78,028,257	234	48,273,550
2000	207	42,692,596	231	50,587,672	394	78,941,529	254	48,946,734
2001	183	43,061,302	240	51017605	362	80,371,681	246	50,068,110
2002	179	43,346,662	279	51,314,670	351	81,508,351	224	50,892,480
2003	178	43,617,202	252	51,631,059	343	82,585,682	242	51,645,340
2004	174	43,819,373	241	51,946,501	335	83,932,308	206	52,455,314
2005	167	44,018,940	240	52,273,671	380	85,380,804	213	53,324,138
2006	160	44,229,025	246	52,619,995	372	86,806,861	255	54,207,322
2007	186	44,457,927	253	52,924,127	371	88,171,923	266	54,995,615
2008	176	44,741,455	225	53,189,240	360	89,446,027	267	55,809,860
2009	175	45,040,112	256	53,441,977	386	90,631,097	217	56,570,762
2010	182	45,254,463	238	53,637,169	420	91,508,374	228	57,118,319
2011	164	45,540,781	248	53,973,947	397	92,862,799	343	58,013,284
2012	164	45,828,012	242	54,198,561	440	94,011,879	332	58,731,490
2013	184	46,050,077	262	54,488,540	427	95,081,366	301	59,419,733
2014	179	46,304,430	301	54,750,576	477	96,395,376	349	60,338,719
2015	168	46,498,349	285	54,979,293	502	97,726,584	383	61,197,807
2016	200	46,500,752	310	55,077,712	590	98,780,134	414	61,793,846
2017	223	46,802,310	331	55,342,525	594	100,014,822	437	62,537,969
2018	213	46,545,043	339	55,521,758	651	101,070,908	465	63,144,281
2019	200	46,494,583	381	55,629,071	670	101,927,428	515	63,617,595
2020	239	46,433,303	459	55,699,847	854	102,997,343	581	64,060,204
2021	290	47,521,188	511	56,137,533	945	103,502,714	715	64,165,640
2022	305	47,614,213	447	56,362,716	920	105,203,667	685	64,669,574
2023	278	47,594,080	479	56,488,381	1007	106,369,144	661	64,964,809

**Table 6:** Age-adjusted mortality rates (AAMRs) with 95% confidence intervals associated with portal hypertension in the United States, stratified by U.S. Census geographic regions (Northeast, Midwest, South, and West), 1999–2023.

Year	Northeast AAMR	Midwest AAMR	South AAMR	West AAMR
1999	0.5 (0.5–0.6)	0.5 (0.5–0.6)	0.5 (0.5–0.6)	0.5 (0.4–0.6)
2000	0.5 (0.4–0.5)	0.4 (0.4–0.5)	0.5 (0.4–0.5)	0.5 (0.5–0.6)
2001	0.4 (0.4–0.5)	0.5 (0.4–0.5)	0.4 (0.4–0.5)	0.5 (0.4–0.6)
2002	0.4 (0.3–0.4)	0.5 (0.5–0.6)	0.4 (0.4–0.5)	0.5 (0.4–0.5)
2003	0.4 (0.3–0.4)	0.5 (0.4–0.5)	0.4 (0.4–0.5)	0.5 (0.4–0.6)
2004	0.4 (0.3–0.4)	0.5 (0.4–0.5)	0.4 (0.3–0.4)	0.4 (0.3–0.5)
2005	0.4 (0.3–0.4)	0.4 (0.4–0.5)	0.4 (0.4–0.5)	0.4 (0.4–0.5)
2006	0.3 (0.3–0.4)	0.4 (0.4–0.5)	0.4 (0.4–0.4)	0.5 (0.4–0.5)
2007	0.4 (0.3–0.4)	0.4 (0.4–0.5)	0.4 (0.3–0.4)	0.5 (0.4–0.6)
2008	0.4 (0.3–0.4)	0.4 (0.3–0.4)	0.4 (0.3–0.4)	0.5 (0.4–0.5)
2009	0.4 (0.3–0.4)	0.4 (0.4–0.5)	0.4 (0.4–0.4)	0.4 (0.3–0.4)
2010	0.3 (0.3–0.4)	0.4 (0.3–0.4)	0.4 (0.4–0.5)	0.4 (0.3–0.4)
2011	0.3 (0.3–0.4)	0.4 (0.4–0.5)	0.4 (0.3–0.4)	0.6 (0.5–0.6)
2012	0.3 (0.3–0.3)	0.4 (0.3–0.4)	0.4 (0.4–0.5)	0.5 (0.4–0.6)
2013	0.3 (0.3–0.4)	0.5 (0.4–0.5)	0.4 (0.4–0.4)	0.5 (0.4–0.5)
2014	0.3 (0.3–0.4)	0.5 (0.4–0.5)	0.4 (0.4–0.5)	0.5 (0.5–0.6)
2015	0.3 (0.3–0.4)	0.5 (0.4–0.5)	0.5 (0.4–0.5)	0.6 (0.5–0.6)
2016	0.3 (0.3–0.4)	0.5 (0.4–0.5)	0.5 (0.5–0.5)	0.6 (0.5–0.7)
2017	0.4 (0.3–0.5)	0.5 (0.5–0.6)	0.5 (0.5–0.5)	0.6 (0.6–0.7)
2018	0.4 (0.3–0.4)	0.5 (0.5–0.6)	0.5 (0.5–0.6)	0.7 (0.6–0.7)
2019	0.3 (0.3–0.4)	0.6 (0.5–0.6)	0.5 (0.5–0.6)	0.7 (0.7–0.8)
2020	0.4 (0.4–0.5)	0.7 (0.6–0.7)	0.7 (0.7–0.8)	0.8 (0.7–0.9)
2021	0.5 (0.4–0.6)	0.8 (0.7–0.8)	0.8 (0.7–0.8)	1.0 (0.9–1.1)
2022	0.5 (0.5–0.6)	0.7 (0.6–0.7)	0.7 (0.7–0.8)	0.9 (0.9–1.0)
2023	0.5 (0.4–0.5)	0.7 (0.6–0.8)	0.8 (0.7–0.8)	0.9 (0.8–1.0)

**Figure 4.**
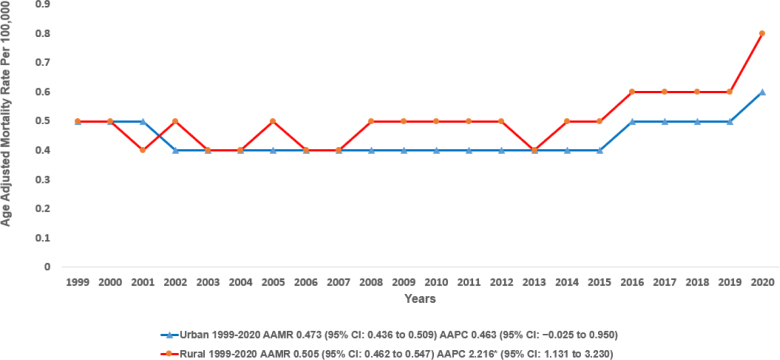
Urban and rural stratified portal hypertension age-adjusted mortality rates per 100,000 adults in the United States, 1999–2020. AAMR = age-adjusted mortality rate, AAPC = average annual percent change, CI = confidence interval.

In urban areas, mortality declined from 1999 to 2003 (0.5 to 0.4; APC = −6.51; 95% CI: −11.53 to −3.68), remained stable from 2003 to 2014 (APC = 0.04; 95% CI: −0.94 to 1.14), and increased significantly from 2014 to 2020 (0.4 to 0.6; APC = 6.21; 95% CI: 4.40 to 9.43; Tables 7 and 8; Fig. 4). In rural areas, mortality remained stable from 1999 to 2013 (APC = 0.15; 95% CI: −3.58 to 1.48), followed by a significant increase from 2013 to 2020 (0.4 to 0.8; APC = 6.48; 95% CI: 2.86 to 17.78).

**Table 7:** Overall deaths associated with portal hypertension by urban and rural populations, United States, 1999–2020.

Year	Urban population	Urban deaths	Rural population	Rural deaths
1999	183,731,694	930	35,353,106	204
2000	185,617,929	893	35,550,602	193
2001	188,798,253	856	35,720,445	175
2002	191,150,095	853	35,912,068	180
2003	193,354,753	835	36,124,530	180
2004	195,794,321	777	36,359,175	179
2005	198,389,174	813	36,608,379	187
2006	200,967,338	857	36,895,865	176
2007	203,448,956	902	37,100,636	174
2008	205,923,317	831	37,263,265	197
2009	208,311,115	831	37,372,833	203
2010	210,033,725	857	37,484,600	211
2011	212,804,208	943	37,586,603	209
2012	215,184,847	963	37,585,095	215
2013	217,407,626	964	37,632,090	210
2014	220,121,808	1055	37,667,293	251
2015	222,697,459	1098	37,704,574	240
2016	224,454,736	1229	37,697,708	285
2017	226,993,727	1314	37,703,899	271
2018	228,517,514	1377	37,764,476	291
2019	229,885,901	1477	37,782,776	289
2020	231,380,941	1735	37,802,256	398

**Table 8:** Age-adjusted mortality rates (AAMRs) with 95% confidence intervals for portal hypertension by urban and rural populations, United States, 1999–2020.

Year	Urban AAMR	Rural AAMR
1999	0.5 (0.5–0.6)	0.5 (0.5–0.6)
2000	0.5 (0.5–0.5)	0.5 (0.4–0.6)
2001	0.5 (0.4–0.5)	0.4 (0.4–0.5)
2002	0.4 (0.4–0.5)	0.5 (0.4–0.5)
2003	0.4 (0.4–0.4)	0.4 (0.4–0.5)
2004	0.4 (0.4–0.4)	0.4 (0.4–0.5)
2005	0.4 (0.4–0.4)	0.5 (0.4–0.5)
2006	0.4 (0.4–0.5)	0.4 (0.3–0.5)
2007	0.4 (0.4–0.4)	0.4 (0.3–0.5)
2008	0.4 (0.4–0.4)	0.5 (0.4–0.5)
2009	0.4 (0.3–0.4)	0.5 (0.4–0.5)
2010	0.4 (0.3–0.4)	0.5 (0.4–0.5)
2011	0.4 (0.4–0.4)	0.5 (0.4–0.6)
2012	0.4 (0.4–0.4)	0.5 (0.4–0.5)
2013	0.4 (0.4–0.4)	0.4 (0.4–0.5)
2014	0.4 (0.4–0.5)	0.5 (0.5–0.6)
2015	0.4 (0.4–0.5)	0.5 (0.4–0.6)
2016	0.5 (0.5–0.5)	0.6 (0.5–0.7)
2017	0.5 (0.5–0.5)	0.6 (0.5–0.6)
2018	0.5 (0.5–0.5)	0.6 (0.5–0.7)
2019	0.5 (0.5–0.6)	0.6 (0.5–0.7)
2020	0.6 (0.6–0.7)	0.8 (0.8–0.9)

Trends were also consistent in direction when comparing crude and AAMRs, as well as multiple cause and underlying cause of death analyses, supporting the robustness of the findings.

## 4. Discussion

The initial decline in overall portal hypertension related mortality is consistent with advances in the management of variceal bleeding, including the widespread adoption of nonselective β-blockers and endoscopic variceal ligation.^[13]^ In contrast, the marked increase observed after 2015 coincides with a growing burden of cirrhosis, which may be related in part to lifestyle factors such as physical inactivity and diets high in carbohydrates and saturated fats, contributing to the rising prevalence of metabolic dysfunction associated steatohepatitis as described in prior population-based studies.^[14]^ Concurrently, the persistent and widespread consumption of alcohol in the United States, previously associated with progression of chronic liver disease and portal hypertension, alongside the rising prevalence of metabolic syndrome and its components including obesity, insulin resistance, type 2 diabetes, and dyslipidemia, represent 2 converging epidemiologic forces that may underlie the observed upward trajectory across all subsequent stratified analyses.^[15]^ The small, not statistically significant drop seen after 2021 could be related to coding or reporting errors associated with COVID-19, improvements in inpatient management, or a combination of both factors.^[16]^ Nevertheless, mortality rates remain higher than those observed during earlier baseline periods, underscoring the continued need for intensified efforts in both prevention and optimized management to reduce the overall disease burden.

Among females, portal hypertension related mortality increased significantly after 2009. This trend coincides with a rising burden of metabolic dysfunction associated with fatty liver disease, including nonalcoholic fatty liver disease (NAFLD) and metabolic dysfunction-associated steatohepatitis, in women, as documented in prior epidemiologic literature.^[17]^ The role of metabolic syndrome components in accelerating progression from hepatic steatosis to advanced fibrosis and cirrhosis may be particularly pronounced in women given sex specific biological vulnerabilities.^[17]^ Delayed diagnosis may also play a role, as prior studies have reported that women with chronic liver disease more frequently present at later stages.^[18]^ Additionally, evidence from the literature suggests that loss of estrogen following menopause may exacerbate hepatic fibrosis and portal pressure progression, potentially increasing susceptibility to advanced liver disease in older women, though this mechanism was not directly accessible in the present study.^[19]^ Women have also been shown to exhibit heightened hepatotoxicity at lower levels of alcohol exposure, and concurrent metabolic and alcohol induced liver injury may collaboratively accelerate fibrosis progression, which may offer a partial explanatory framework for the observed sex specific trends.^[20]^

Among males, portal hypertension related mortality demonstrated a nonsignificant decline until 2013, followed by a statistically significant upward trend thereafter. Beyond the shared burden of metabolic liver disease and alcohol associated liver disease, several male specific risk factors may further explain this pattern. Historically, men have had higher rates of heavy alcohol consumption, which has been associated with earlier progression to cirrhosis and portal hypertension.^[21]^ In recent years, population level data have documented rising levels of high risk drinking, binge drinking, and alcohol use disorder particularly among middle aged and older men, which have been linked to increasing rates of alcohol-related liver disease related cirrhosis and portal hypertension mortality.^[22]^ Additionally, men constituted a substantial proportion of individuals infected with the hepatitis C virus (HCV) in prior decades; as this cohort ages, the cumulative hepatic injury associated with long standing HCV infection has been reported to translate into higher rates of advanced fibrosis, cirrhosis, and portal hypertension, despite advances in antiviral therapy.^[23]^ Although direct acting antivirals have reduced the incidence of HCV related liver disease, many men had already developed established cirrhosis at the time of treatment, which prior studies suggest limits the potential for reversal of portal hypertension and long-term mortality reduction.^[23]^ Furthermore, men have been reported to be more likely to present with decompensated cirrhosis and its complications including refractory ascites, variceal hemorrhage, spontaneous bacterial peritonitis, and hepatic encephalopathy, all of which are associated with increased mortality in the existing literature.^[24]^ Finally, higher rates of smoking, suboptimal dietary patterns, and lower utilization of preventive healthcare services among men have been previously described as factors that may accelerate disease progression and contribute to excess mortality.^[25,26]^

Racial and ethnic disparities were evident throughout the study period. Hispanic individuals consistently demonstrated the highest burden of portal hypertension-related mortality, followed by White individuals, whereas Black individuals exhibited comparatively lower rates. The initial decline in portal hypertension mortality between 1999 and 2003 may be attributable to improvements in the management of portal hypertension and its complications. During the 1990s and early 2000s, advances such as endoscopic variceal band ligation, transjugular intrahepatic portosystemic shunt, and improved critical care management substantially reduced mortality associated with variceal bleeding and decompensated cirrhosis.^[27]^ These therapeutic developments likely benefited all racial and ethnic groups and contributed to the early downward trend observed in our analysis.

The subsequent period of relative stabilization from 2003 to 2013 may reflect a balance between therapeutic advances and the growing burden of chronic liver disease. Among Hispanic populations, the prevalence of metabolic syndrome remained substantially higher than that of non-Hispanic White and Black populations. NHANES data demonstrated the prevalence of metabolic syndrome at 31.9% among Hispanics, compared with 23.8% among non-Hispanic Whites and 21.6% among African Americans.^[28]^ Hispanic individuals also exhibit the highest intraperitoneal and hepatic fat content, greater insulin resistance, and a significantly higher prevalence of NAFLD, with hepatic steatosis affecting approximately 45% of the Hispanic population.^[28]^ Furthermore, Hispanic patients with NAFLD are more likely to demonstrate advanced fibrosis, hepatocyte ballooning, and inflammatory activity, suggesting accelerated progression to cirrhosis and clinically significant portal hypertension.^[28]^ Concurrently, chronic HCV infection remained disproportionately prevalent among Hispanic and Black populations compared with Whites, further contributing to chronic liver disease burden during this period.^[28]^ Genetic susceptibility may also contribute, as the PNPLA3 risk allele, which is associated with hepatic steatosis, fibrosis progression, and alcohol-related liver injury, is more prevalent among Hispanics.^[29]^ In addition, Hispanics exhibit the highest rates of nonalcoholic steatohepatitis and advanced fibrosis, placing them at greater risk of cirrhosis, portal hypertension, and hepatocellular carcinoma.^[29]^ The marked increase in portal hypertension mortality after 2013 likely reflects the convergence of several adverse epidemiologic trends. First, alcohol-associated liver disease emerged as a major driver of cirrhosis-related mortality in the United States.^[29]^ Studies have consistently shown that alcoholic liver disease is the predominant cause of cirrhosis among White individuals.^[29]^ National data indicate that alcohol consumption increased substantially during the past 2 decades, with additional acceleration during the COVID-19 pandemic.^[29]^

In contrast, Black individuals demonstrated lower portal hypertension mortality rates throughout the study period. The rate of metabolic syndrome is lower among Black men (22%) than among White men (33%), and Hispanic men (25%).^[30]^ Although chronic HCV infection has historically been more prevalent among Black individuals, the lower prevalence of NAFLD, lower rates of severe steatohepatitis, and reduced burden of alcohol-related cirrhosis may have attenuated the overall increase in portal hypertension mortality compared with Hispanic and White populations.^[31]^ Collectively, these findings suggest that the racial and ethnic disparities observed in portal hypertension mortality from 1999 to 2023 largely mirror differences in the epidemiology of chronic liver disease in the United States.

Region specific analyses revealed substantial heterogeneity in portal hypertension related mortality trends across the United States. The Northeast experienced a significant decline in mortality through 2014, a pattern that is consistent with previously reported advantages in access to specialty care and earlier regional adoption of evidence-based cirrhosis management strategies.^[32]^ However, this decline was followed by a significant increase thereafter, which may reflect the growing burden of metabolic liver disease, an aging cirrhosis population, and a potential catch up effect as improved survival allows accumulation of decompensated disease.^[33]^ Despite this recent rise, the Northeast continued to exhibit the lowest absolute mortality rates among all regions.^[33]^ In contrast, the South demonstrated the steepest increase in mortality beginning in 2013, representing the largest and fastest growing regional burden in the present analysis. This trend coincides with a documented high regional prevalence of obesity, diabetes, and metabolic syndrome; elevated rates of alcohol associated liver disease; and pronounced socioeconomic disparities that have been associated with limited access to specialized hepatology care in prior literature.^[34]^ The Midwest showed a significant upward trend in mortality starting around 2010, consistent with the national epidemiologic shift toward metabolic and alcohol related liver disease, further compounded by rising rates of obesity and alcohol use in rural and semi urban populations specific to this region.^[35]^ Similarly, the West experienced a significant increase in mortality after 2010, alongside the presence of large immigrant populations who have been reported to present with more advanced liver disease in prior studies, adding a region-specific demographic dimension to the broader metabolic trends already described.^[36]^ Absolute mortality rates in the West were higher than those observed in the Northeast and Midwest, underscoring the regional variability in disease burden identified in the present analysis.

Urban rural stratified analyses demonstrated distinct temporal patterns in portal hypertension related mortality. Urban areas showed earlier improvements through 2014, followed by a more gradual increase through 2020. The initial decline in urban populations is consistent with previously reported advantages in access to tertiary care centers, higher levels of health education, and greater adherence to evidence based therapies, including early adoption of endoscopic variceal ligation and nonselective β-blockers.^[37]^ The subsequent rise after 2014 likely reflects the same converging pressures of metabolic liver disease burden and aging cirrhotic populations described in prior subsections.^[38]^ In contrast, rural populations exhibited a delayed but steeper increase in mortality beginning around 2013, indicating worsening disparities through 2020. Notably, while urban and rural AAMRs were comparable in 1999 (both approximately 0.5 per 100,000), by 2020 rural mortality rates exceeded urban rates (0.8 vs 0.6 per 100,000), corresponding to an absolute difference of 0.2 and a relative increase of approximately 1.3-fold. This divergence is consistent with the compounded effect of the national metabolic and alcohol related disease burden, alongside rural specific barriers including limited access to hepatology care, delayed diagnosis, fewer screening opportunities, and more advanced disease at presentation in rural communities.^[32,34]^ Collectively, these observations suggest a disproportionate increase in portal hypertension related mortality in rural populations, though direct causal attribution cannot be established from mortality data alone.

## 5. Limitations and future directions

Although this study provides important insights into national mortality trends associated with portal hypertension, several limitations merit consideration. First, the analysis relies on death certificate data from the CDC WONDER database, which are inherently subject to misclassification and underreporting. Portal hypertension is rarely recorded as the underlying cause of death and is more often documented as a secondary or contributing condition, leading to probable underestimation of its true mortality burden. Variability in ICD coding practices across states, institutions, and certifying physicians may further affect the accuracy of cause-of-death attribution, particularly in patients with advanced cirrhosis and multiple competing comorbidities.

Second, CDC WONDER lacks granular clinical data, including portal pressure measurements, disease etiology, severity of portal hypertension, cirrhosis stage, laboratory parameters, and treatment-related variables such as nonselective beta-blocker use, endoscopic surveillance, trans jugular intrahepatic portosystemic shunt placement, or liver transplantation status. The absence of this data limits the ability to stratify mortality risk by disease phenotype, severity, or adherence to guideline-directed therapies. Additionally, comorbid conditions that frequently influence outcomes – such as alcohol use disorder, metabolic dysfunction, cardiovascular disease, infections, malnutrition, and sarcopenia – cannot be reliably assessed in mortality-only datasets.

Third, this study captures only fatal outcomes and does not account for nonfatal clinical trajectories, including hospitalizations for variceal bleeding, ascites, hepatic encephalopathy, spontaneous bacterial peritonitis, or acute-on-chronic liver failure. Consequently, while the findings characterize mortality patterns, they do not capture the broader morbidity burden or healthcare utilization associated with portal hypertension. Furthermore, although place of death is recorded, information regarding longitudinal care settings, access to hepatology services, intensity of outpatient management, or transitions to palliative or hospice care is unavailable, limiting insights into healthcare delivery and end-of-life decision-making.

Fourth, mortality records do not distinguish whether deaths were directly attributable to portal hypertension complications, progressive liver failure, infections, renal dysfunction, or extrahepatic causes. Without linked person level datasets such as medicare claims, healthcare cost and utilization project hospitalization data, or transplant registries, it is not possible to determine whether observed mortality trends are driven by increasing disease incidence, worsening clinical outcomes, disparities in access to care, or changes in documentation of liver related conditions over time. Additionally, structural and socioeconomic determinants, including race and ethnicity, rural–urban residence, insurance status, and regional availability of specialized liver care, may contribute to observed disparities but could not be fully explored within the constraints of the dataset.

Finally, the Joinpoint regression approach assumes log-linear trends between inflection points and may oversimplify complex, nonlinear mortality patterns influenced by evolving diagnostic practices, changes in ICD coding, public health interventions, and major healthcare disruptions such as the COVID-19 pandemic.

Future research should leverage linked, longitudinal datasets integrating clinical, administrative, and laboratory information to better delineate causal pathways between portal hypertension and mortality. Prospective studies evaluating systematic screening for clinically significant portal hypertension, adherence to prophylactic strategies, and timely management of complications are needed to identify modifiable drivers of mortality. Additionally, investigations focused on health equity, telemedicine-based hepatology care, and policy-level interventions aimed at improving access to early diagnosis and specialized treatment will be critical for reducing the population-level burden of portal hypertension and its complications.

## 6. Conclusion

Portal hypertension-related mortality in the United States initially declined with the adoption of evidence-based therapies but has risen steadily since the mid-2010s, driven by the growing burden of metabolic dysfunction-associated liver disease, persistent alcohol-associated liver disease, and an aging cirrhotic population surviving longer yet progressing to decompensation. Significant disparities by sex, race/ethnicity, region, and urban–rural status underscore inequities in risk factor exposure, disease recognition, and access to specialty care, with particularly steep increases observed among women, rural populations, residents of the Southern United States, and Hispanic or Latino individuals, who consistently demonstrated the highest mortality burden throughout the study period, alongside a sustained rise among White and Black or African American individuals reflecting the converging epidemiologic forces of metabolic and alcohol-associated liver disease. Reducing mortality will require a shift toward proactive, prevention-focused strategies, including early identification of chronic liver disease through targeted screening of high-risk individuals, aggressive management of metabolic and alcohol-related risk factors, culturally and linguistically appropriate care models to address barriers disproportionately affecting Hispanic populations, and improved continuity of care through structured follow-up, multidisciplinary cirrhosis clinics, and tele-hepatology models to reach underserved communities. Strengthening preventive care pathways and ensuring timely surveillance and intervention may help mitigate late-stage decompensation, narrow existing disparities across racial and ethnic groups, and ultimately reduce the rising mortality burden associated with portal hypertension.

## Author contributions

**Conceptualization:** Raymond Haward.

**Data curation:** Raymond Haward, Pooja Prasad.

**Formal analysis:** Raymond Haward.

**Investigation:** Raymond Haward, Harkanwar Sandhu, Pooja Prasad.

**Methodology:** Harkanwar Sandhu, Rachel Haward, Pooja Prasad.

**Resources:** Rachel Haward.

**Software:** Nikhil Duseja.

**Supervision:** Mobeen Abid, Iswariya Mani.

**Validation:** Swathi Deepak Kulkarni.

**Visualization:** Nikhil Duseja, Jai Kumar Rajavoor Muniswamy.

**Writing – original draft:** Raymond Haward, Nikhil Duseja, Rachel Haward, Mobeen Abid, Iswariya Mani, Pooja Prasad.

**Writing – review & editing:** Het Hirpara, Harkanwar Sandhu, Swathi Deepak Kulkarni, Mir Mehdi Hussain, Newton Ashish Shah, Jai Kumar Rajavoor Muniswamy.
